# RETRACTED: Aldakheel et al. Employing of Curcumin–Silver Nanoparticle-Incorporated Sodium Alginate-Co-Acacia Gum Film Hydrogels for Wound Dressing. *Gels* 2023, *9*, 780

**DOI:** 10.3390/gels10060383

**Published:** 2024-06-05

**Authors:** Fahad M. Aldakheel, Dalia Mohsen, Marwa M. El Sayed, Mohammed H. Fagir, Dalia K. El Dein

**Affiliations:** 1 Department of Clinical Laboratory Sciences, College of Applied Medical Sciences, King Saud University, Riyadh 11433, Saudi Arabia; faldakheel@ksu.edu.sa; 2 Clinical Laboratory Sciences Program, Inaya Medical College, Riyadh 12211, Saudi Arabia; husseinfagir@inaya.edu.sa (M.H.F.); dkmohammed@inaya.edu.sa (D.K.E.D.); 3 Microbiology Department, National Research Centre, Giza 12622, Egypt; 4 Chemical Engineering and Pilot Plant Department, National Research Centre, Giza 12622, Egypt; dr.marwameid@gmail.com

The *Gels* Editorial Office retracts the article, “Employing of Curcumin–Silver Nanoparticle-Incorporated Sodium Alginate-Co-Acacia Gum Film Hydrogels for Wound Dressing” [1], cited above.

Following publication, concerns were brought to the attention of the publisher regarding unattributed overlap with a previously published article from a different authorship group.

Adhering to our complaint’s procedure, an investigation was conducted by the Editorial Office and Editorial Board that confirmed a significant overlap of methodology, data and a figure (Figure 1) between this article [1] and a previously published article [2], without appropriate citation. As a result, the Editorial Office, the Editorial Board and the authors have decided to retract this paper as per MDPI’s retraction policy (https://www.mdpi.com/ethics#_bookmark30, accessed on 8 May 2024) and in line with the Committee on Publication Ethics retraction guidelines (https://publicationethics.org/retraction-guidelines, accessed on 8 May 2024).

This retraction was approved by the Editor-in-Chief of the journal *Gels*.

The authors agree to this retraction.

## Figures and Tables

**Figure 1:**
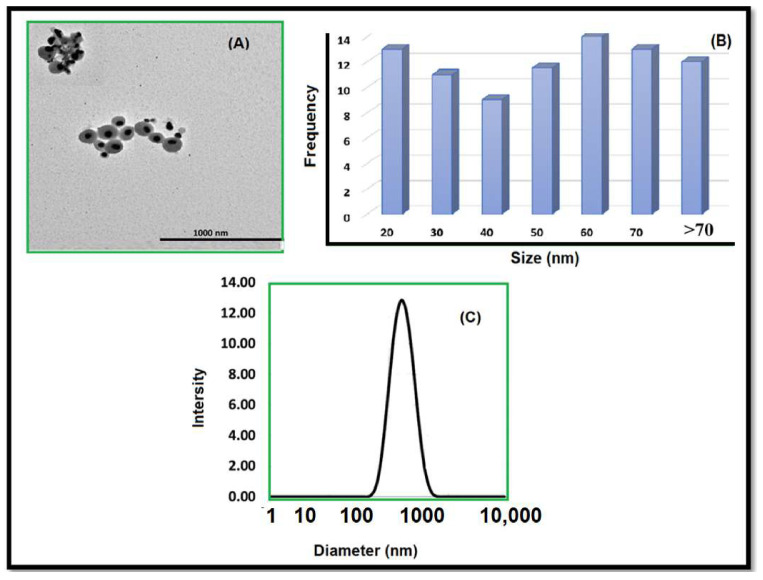
(**A**) TEM characterization of AgNPs, (**B**) size distribution as measured by TEM analysis and calculated with 100 nanoparticles, and (**C**) DLS data of AgNPs with size distribution.
